# Effects of treatment with three types of varnish remineralizing agents on the microhardness of demineralized enamel surface

**DOI:** 10.4317/jced.55611

**Published:** 2019-07-01

**Authors:** Fahimeh Kooshki, Sahar Pajoohan, Sanaz Kamareh

**Affiliations:** 1 Assistant Professor, Department of Pediatric Dentistry, School of Dentistry, Shahid Beheshti University of Medical Sciences, Tehran, Iran; 2 Private practice, Tehran, Iran; 3 Postgraduate student of Pediatric dentistry, Department of Pediatric dentistry, School of Dentistry , Shahid Beheshti University of Medical sciences, Tehran, Iran

## Abstract

**Background:**

Remineralization of incipient caries is one of the goals in dental health care ,especially in pediatric dentistry. The present study aimed at comparing the effects of MI varnish (3M (United states)) , Nano paste( FGM(Brezil) ), 5% sodium fluoride varnish) DuraphatColgate (united states) ) on remineralization of enamel lesions.

**Material and Methods:**

In this *in-vitro*study, 60 intact human pre-molars, were randomly allocated to four groups of 15. Baseline surface microhardness in three points in the center of the polished area was measured. After two days of immersion in demineralizing solution, microhardness of all samples was measured. Afterward, groups 1-3 under-went treatment with MI varnish, nano paste, 5% sodium fluoride varnish and then again microhardness was measured. The results were analyzed by one-way analysis of variance (ANOVA), repeated measures ANOVA, and Bonfreni table was used.

**Results:**

Duraphat varnish in comparison with control group, significantly increased surface microhardness and in comparison with Nano and MI paste varnish groups significant differences was shown between groups. (*P*< 0.05). MI paste varnish and Nano paste similary showed more increases in surface microhardness in comparison with Duraphat varnish and control groups (P≈1).

**Conclusions:**

According to the results of this study, all three varnishes, Duraphat , MI paste and Nano paste increase the enamel surface microhardness and remineralization of incipient caries. MI paste and Nano paste compared to Duraphat Varnish, significantly showed more increases in enamel surface microhardness but Nano paste and MI paste were almost the same.

** Key words:**CPP-ACP, Nano varnish, fluoride varnish, microhardness, demineralization, remineralization.

## Introduction

Dental caries is a chronic, multifactorial, transmissible, infectious disease which occurs when the presence of acids on the tooth-plaque interface and leads to a shift in the demineralization/remineralization equilibrium favoring a net demineralization of the enamel (1,2). While caries is a preventable disease that has witnessed a decline in most developed countries in recent years, it continues to remain a major health problem, especially among young children (3).The use of fluoride in water supplies, toothpastes and professional topical fluoride therapy is considered to be a key means of preventing dental caries (4).

The anti-cariouse effectiveness of topical fluoride therapy has been well established, and professional topical fluoride applications are commonly used to arrest the progression of active lesions (5). Its mechanisms included reduction of acid production by microorganisms, inhibition of intracellular and extracellular enzymes, and replacement of hydroxide ions in hydroxyapatite with fluoride ions (resulting in acid resistant fluoroapatite crystals) (6).

Fluoride varnish is a fluoride-concentrated form ,which is used as a type of topical fluoride treatments. Due to its easiness and similar efficiency with fluoride gel system, varnishes especially in Pre-school children is recommended (7). Due to its sticky nature, it can remain in contact with the tooth surface for several hours, which will prevent new carious lesions and extension of previous caries (8). Fluoride Varnish is a safe way to protects the teeth and provides the highest and safest possible fluoride concentration (8,9). In young children, that there is a possibility of fluoride ingestion after fluoride therapy with gels and mouth washes , using fluoride varnish seems to be a good way for topical fluoride therapy (10). However, new tooth remineralizing agents have been developed, including compounds with the additional or synergistic effects of fluoride to enhance the remineralization and improve the mechanical properties of the demineralized surfaces, such as phosphopeptides from milk protein casein and nano- hydroxyapatite (nano-HAP) (11-16).

Casein phosphopeptides (CPPs) stabilize nanoclusters of amorphous calcium phosphate (ACP) in a metastable solution (11). CPPs bind to the ACPs nanoclusters in the supersaturated solutions, preventing the precipitation of calcium and phosphate (11). The casein phosphopeptide– amorphous calcium phosphate (CPP-ACP) complex also acts as a reservoir for storing bioavailable calcium and phosphate and maintains the solution supersaturated, hence reinforcing remineralization (11). Several studies have demonstrated the efﬁcacy of CPP-ACP technology in enamel and dentin demineralization inhibition, and remineralization promotion (11-14,17). The CPP-ACP complex is commercially available in mousse or paste form, and the product MI Paste Plus (GC America Inc., Alsip, Ill., USA) contains fluoride (900 ppm)and CPP-ACP.

Nano-HAP is considered one of the most bioactive and biocompatible materials; it has potential effect of remineralizing initial enamel caries under dynamic pH-cycling conditions (15,16). The Nano-HAP paste contains calcium nanophosphate organized in a crystalline form of hydroxyapatite and 9,000 ppm of fluoride (Desensibilize Nano P, FGM, Joinville, Santa Catariuna, Brazil). This agents indicated for desensitization and/or remineralization of the enamel and is commercially available for professional use (15).

Considering the limited research on the effectiveness of this newly introduced product, the present study aimed at comparing the effects of MI varnish (3M(United states)), Nano paste(FGM(Brezil)) and Duraphat (Colgate(united states)) on remineralization of enamel lesions. As enamel surface microhardness is related with its minerals contents (18), this index was measured as a criterion to determine enamel demineralization and remineralization.

## Material and Methods

This *in vitro* study was performed on 60 intact human pre-molars extracted for orthodontic reasons (not only for study).The power of the study was kept at 0.80 and confidence level at 95%. Estimated sample size 15 in each experimental group (9) . The teeth were immersed in Normal Salin post-extraction and solution was renew every two weeks and maintained at room temperature for about six months. Premolars were only selected if there were no signs of cavity, restoration, crack, abrasion, or hypoplasia. The contaminants were removed by a hand medium tooth brush (Jordan- Norway) and then cleaned with H2O2 (3%), then samples were kept in Normal Salin at room temperature.

In the next stage, tooth roots were cut by a diamond disc (D&Z, Ber-lin, Germany) andPoly styrene was used to mount each tooth crown. In order to smooth tooth surfaces and increase the accuracy of microhardness measurements, the buccal surfaces were polished with a silicon carbide paper (800,1000,2000 grit size, USA).

The samples were randomly allocated to four groups of 15. Baseline surface microhardness in three points in the center of the polished area was measured using a Vickers Hardness Testing Machine (INNOVA TEST, Netherlands) with a force of 200 N and length of 10 s (6).

Caries-like lesions on the enamels were produced by incubating the samples at 37ºC for two days (48 hours). The demineralizing solution contained NaCl (2/9 g), CaCl2 (0/12g), NaH2PO4 (0/13g), NaF (5cc)(100 ppm), NaN3 (5cc) (2% ww), Acetic acid (1/5 cc). The pH of the solution was maintained at 4.5 . The solution was renewed daily to prevent the accumulation of materials produced by demineralization and the consequent pH change. After 48 hours, the surface of each sample was washed with a syringe containing artificial saliva(NaCl (2/9 g), CaCl2 (0/12 g), NaH2PO4 (0/13g), NaF (5cc) (100ppm), NaN3 (5cc) (2% ww)). They were then placed in containers containing artificial saliva and their microhardness was remeasured.

Then, all samples were air-dried for 30 s and surface treatments were performed. In the first group, 5% sodium fluoride varnish) DuraphatColgate(united states) (was left on the surface of the samples for one minute according to the manufacturer’s instructions. Following the immersion of the samples in distilled water for 24 hours and incubated at 37ºC (HERAcell 150i).

Then, the remaining fluoride varnish was removed by using a No. 15 scalpel blade. Finally, the samples’ surface was washed with a syringe containing artificial saliva. A similar procedure was followed for the second and third groups. However, surface treatment was conducted by covering the samples with nanopaste ( FGM(Brezil)) in group 2 and with MI varnish (3M(United states)) in group 3.

To simulate the oral environment, the samples were washed and immersed in pH cycle for 24 hours. In every cycles, demineralizing solution was applied for 3 hours and then the samples were washed with distilled water and were immersed for 30 minutes in distilled water. Then remineralizing solution was applied for 24 hours and again the samples were washed with distilled water and were immersed for 30 minutes in distilled water. The same routine was repeated every day for ten days.

After surface treatments, microhardness of all samples was reassessed similar to first and second measurements. Tests were done with a technician who was blinded to samples.

**Figure 1:**
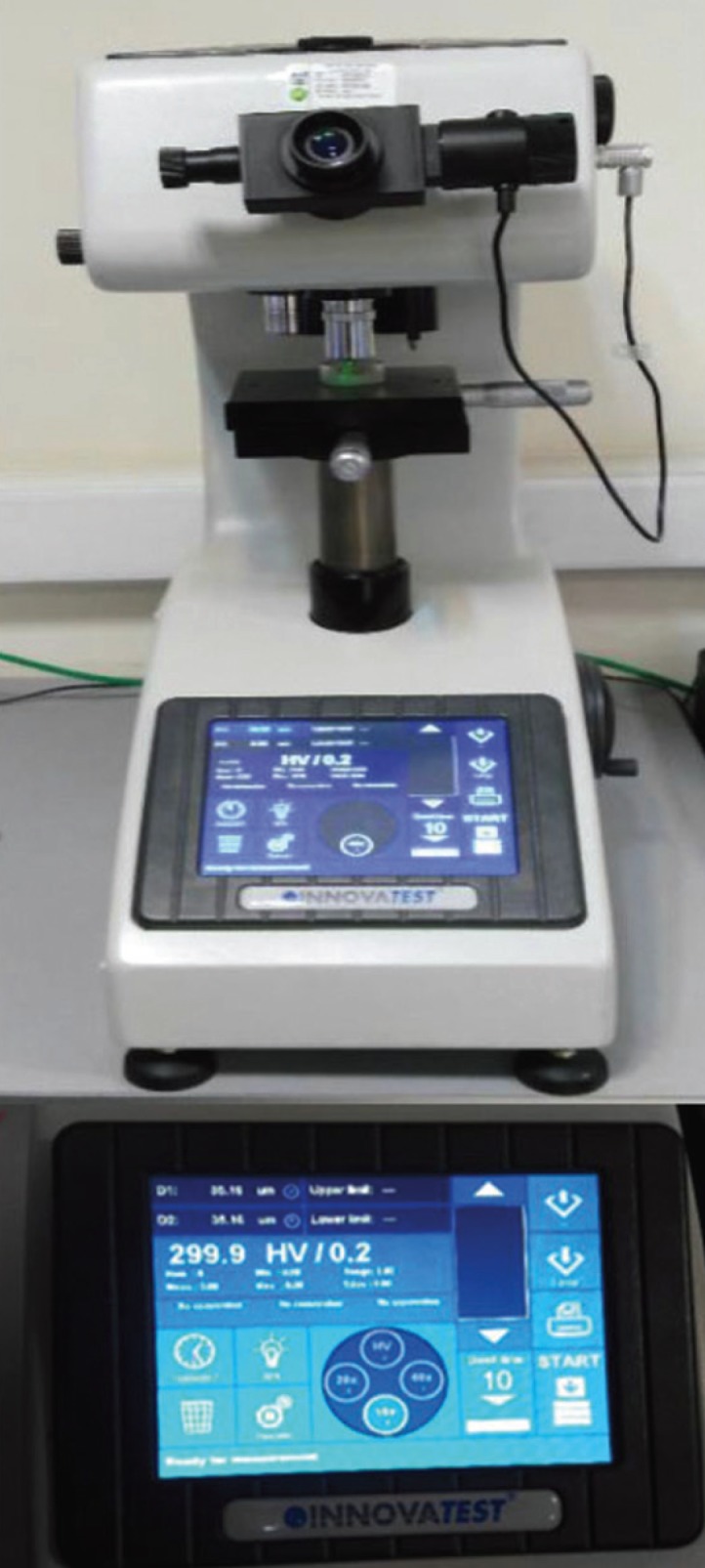
Vickers Hardness Testing Machine (INNOVA TEST, Netherlands).

The mean surface microhardness of all samples was recorded in Vickers hardness number (VHN) in the prepared forms (Fig. 1)

One-way analysis of variance (ANOVA) was applied to compare the mean surface microhardness values at baseline, after primary demineralization, after treatment regimens. Intragroup comparisons of the mean surface microhardness values at different time intervals were performed using repeated measures ANOVA. In addition, Bonfreni table was used for pairwise comparisons of the groups in terms of the mean surface microhardness. All analyses were conducted in SPSS for Windows 22.0 (SPSS Inc., Chicago, IL, USA).

## Results

The mean of surface microhardness values in all groups are presented in  Table 1.

**Table 1:**
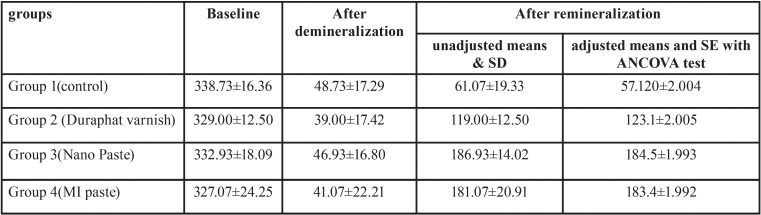
The means and SD surface microhardness( baseline, after demineralization and after remineralization) values in all groups and adjusted mean and SE with ANCOVA test after remineralization.

Evaluation of distribution of microhardness variables before and after demineralization and after remineralization between different groups revealed that, due to the proximity of the mean and median values, the distribution of the microhardness variable in all stages was normal (*P*< 0.05). Therefore, the conditions for using parametric tests were established.

One-way ANOVA results indicated no significant differences in the mean baseline (*p*=0.323) and after two days of demineralization microhardness (*P* =0.429 ), but significant differences was observed in microhardness after remineralization process (*P*< 0.05).

**Figure 2:**
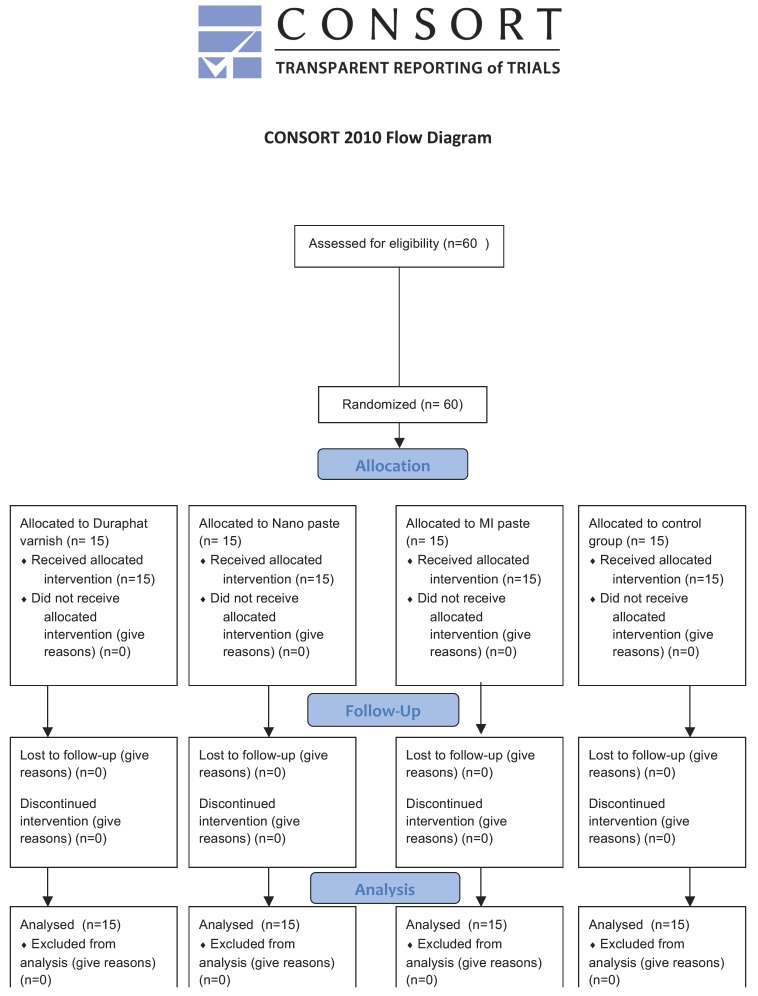
CONSORT Flow Diagram.

Considering that the microhardness rate after demineralization affect our results after the remineralization, ANCOVA analysis was used for neutralization of the effect of this variable (microhardness rate after demineralization). Figure 2 shows the moderated averages after the remineralization in each of the four groups, which is the highest in the Nano group followed by, MI paste group , Durphat Varnish and ultimately the control group.

On the other hand, pairwise comparisons by Bonferroni test based on ANCOVA results, revealed that Duraphat varnish significantly increased surface microhardness and in comparison with Nano and MI paste varnish groups significant difference was shown between groups (*P*< 0.05). MI paste varnish and Nano paste similarly showed higher increased surface microhardness in comparison with Duraphat varnish and control groups (P≈1).

## Discussion

The prevention of dental caries is more important than its treatment. Rapid and progressive carious lesions in infants and young children, called early child-hood caries, is a challenge for pedodontists as it usually requires treatment under general anesthesia. Moreover, adaptation problems, hormonal changes, and extreme use of carbohydrates, e.g. sweets and snacks, increase the risk of caries in adolescents (19) .

Dental caries prevention strategies in children and adolescents can stop incipient carries and remineralize the damaged dental surface. In fact, remineralization of the primary decay and white spots in enamels with preventive materials will in turn decelerate or prevent cavity development and preserve the dental structure . Specifically-designed treatment regimens have been proven to remineralize initial enamel lesions and increase their acid resistance (19).

According to the relationship between mineral content and enamel surface microhardness, it can be used as criterian to assess the efficacy of various remineralizing materials in stopping the demineralization process and reversing lesions. Accordingly, and similar to previous researches ,the mean surface microhardness of all samples at baseline was about 300-350 VHN. However, after the first demineralization it was lower than other studies ( about 40-60 VHN) , which might be due to differences between demineralizating solution . In other words, although enamels with primary lesions manifested an even surface, their mineral content, and thus microhardness, was very lower compared to an intact enamel (6,13,20) .

In the present study, we treated artificial caries with Duraphat varnish, MI paste, and Nano paste, respectively. This study showed that microhardness in all three groups were higher than control group . Nano paste and MI paste showed more increases in microhardness, and Nano paste was slightly more effective than MI paste . However, the difference was not significant.

Salehzadeh *et al.* (6), revealed that one-time professional application of 5% sodium fluoride varnish (DuraShield®, Sultan Health Care, USA) , CPP-ACP (GC Tooth Mousse®, GC, Japan) ,Remin Pro® (VOCO, Germany) , failed to cause remineralization or increase enamel microhardness. Moreover, despite the slight increase in microhardness following the application of CPP-ACP, the three regimens had no significant difference in this regard. Since insufficient exposure time could have been responsible for this finding, repeated application of the mentioned substances over shorter intervals might lead to more favorable results.

The mechanism of action of these materials can be briefed as follows.

Presence of fluoride ions in oral cavity causes the precipitation of fluorapatite from existing calcium and phosphate ions in saliva. The increased PH will then lead to formation of larger acid-resistant crystals containing fluoride (fluorohy-droxyapatite). Consequently, a strong surface layer will develop ,remineralization will be promoted, and finally, the resistance to demineralization will be increased (13).

On the other hand, casein phosphate which presents in CPP-ACP stabilizes calcium and phosphate and facilitates the formation of calcium phosphate nano-complexes on tooth surface. These compounds will act as a source of minerals for remineralization process. In fact, the insoluble form of calcium phosphate becomes soluble in presence of casein phospholipids. Subsequently, amorphous calcium phosphate is formed and localized on tooth surface, and act as a source of calcium and phosphate ions. It helps calcium and phosphate ions to displace deep into lesions through the porous layer on the white spots and to encourage the remineralization of enamels crystals. This material also could rapidly turn into hydroxyapatite which is then deposited on tooth surface (21-23) .

A number of studies have assessed the beneficial effects of Fluoride Varnish on the remineralization of incipient caries. Results of these Studies have shown that microhardness of demineralized enamel due to caries , will increases after application of different concentrations of fluoride varnish.(24-26)

Pithon *et al.*, concluded that the MI paste in remineralizing incipient caries is more effective than Duraphat varnish, that is similar to the results of present study (27).

Huang *et al.* (28), have shown that there is no difference in effectiveness of TCP varnishes, conventional colgate varnishes and common household methods on the spots after orthodontics treatments. According to clinical nature of this study and using intraoral photographs, it seems that assessment of microhardness (present study) is more reliable.

Some previous studies have also suggested surfaces treated with fluoride and CPP-ACP to be more resistant than the intact enamel (29).

The current study design was limited by the simulation of oral cavity conditions. Further studies are needed to compare the effects DIAGNOdent with the mentioned materials through the measurement of microhardness in subsurface layer of treated lesions. In addition, future studies are recommended to measure the depth of lesions under a polarized light microscope before and after treatments.

## Conclusions

According to the results of this study, all three varnishes, Duraphat , MI paste and Nano increase the enamel surface microhardness and remineralization of incipient caries. MI paste and Nanopaste compared to Duraphat Varnish, significantly showed more increases in enamel surface microhardness but Nano paste and MI paste were almost the same.
